# Severe Acute Pancreatitis as an Index Clinical Manifestation of Parathyroid Adenoma

**DOI:** 10.7759/cureus.2445

**Published:** 2018-04-07

**Authors:** Tagore Sunkara, Megan E Caughey, Prashanth Rawla, Krishna Sowjanya Yarlagadda, Vinaya Gaduputi

**Affiliations:** 1 Internal Medicine/gastroenterology, The Brooklyn Hospital Center, Affiliate of the Mount Sinai Hospital. 121 Dekalb Avenue, Brooklyn, Ny 11201; 2 Internal Medicine, New York Institute of Technology College of Osteopathic Medicine, Old Westbury, Ny; 3 Department of Internal Medicine, Memorial Hospital of Martinsville and Henry County, 320 Hospital Drive Martinsville, Va 24115; 4 Department of Internal Medicine, Southwest Community Health Center, 46 Albion Street, Bridgeport, Ct 06605; 5 Department of Internal Medicine, SBH Health System, 4422 Third Ave, Bronx, Ny 10457

**Keywords:** pancreatitis, hyperparathyroidism, hypercalcemia, parathyroid sestamibi scan, abdominal pain, calcium

## Abstract

Hypercalcemia due to primary or secondary hyperparathyroidism is a rare and obscure cause of acute pancreatitis. Although a rare occurrence to begin with, hyperparathyroidism commonly manifests with symptoms of hypercalcemia. Thus, it would reason that a patient might develop pancreatitis by way of hypercalcemia due to primary or secondary hyperparathyroidism. We present a case of an 88-year-old female with acute pancreatitis and only after an extensive work-up, was it determined that her severe acute pancreatitis resulted from primary hyperparathyroidism caused by a left parathyroid adenoma.

## Introduction

Acute pancreatitis is most commonly diagnosed in a patient who meets two of the three following criteria: 1) epigastric abdominal pain that radiates to the back, 2) a three-fold increase in serum lipase or amylase, and 3) findings on cross-sectional abdominal imaging consistent with acute pancreatitis. The most common causes of acute pancreatitis are gallstones (30-60%), alcohol (15-30%), post Endoscopic Retrograde Cholangiopancreatography (ERCP) pancreatitis(5-10%), and hypertriglyceridemia (1.3-3.8%) [1]. Hypercalcemia resulting from hyperparathyroidism that then induces acute pancreatitis is estimated to occur at a prevalence of 1.5-7% [2]. However, a study involving 1475 patients with acute pancreatitis found that only 5 of these patients also had hyperparathyroidism, making the prevalence closer to 0.4% of cases. Therefore, it is an uncommon condition that is thought to develop because high serum calcium levels deposit in the pancreatic ducts in hyperparathyroidism, which then activates the pancreatic enzymes, causing acute pancreatitis [3]. Here, we present the unique instance of a left parathyroid adenoma producing a hypercalcemic state, which subsequently led to the development of acute pancreatitis in an elderly woman [Sunkara T, Caughey ME, Culliford A, Gaduputi V: Severe acute pancreatitis as the index clinical manifestation of parathyroid adenoma in an elderly patient. Program No. P901. World Congress of Gastroenterology at ACG2017 Meeting Abstracts. Orlando, FL: American College of Gastroenterology].

## Case presentation

An 88-year-old woman with past medical history of hypertension, hyperlipidemia, and nephrolithiasis, presented to the Emergency Department complaining of sudden onset of sharp epigastric pain and multiple episodes of non-bloody, non-bilious vomiting of two-day duration. Pain was 9/10 in severity, radiating to the back with no exacerbating or relieving factors. The patient denied any associating symptoms other than vomiting. The patient was afebrile with blood pressure of 138/78, heart rate of 85 and oxygen saturation of 94% on room air. Physical examination elicited epigastric and left upper quadrant tenderness with no guarding or rigidity. The patient denied alcohol consumption or having taken any medications known to cause pancreatitis.

**Figure 1:**
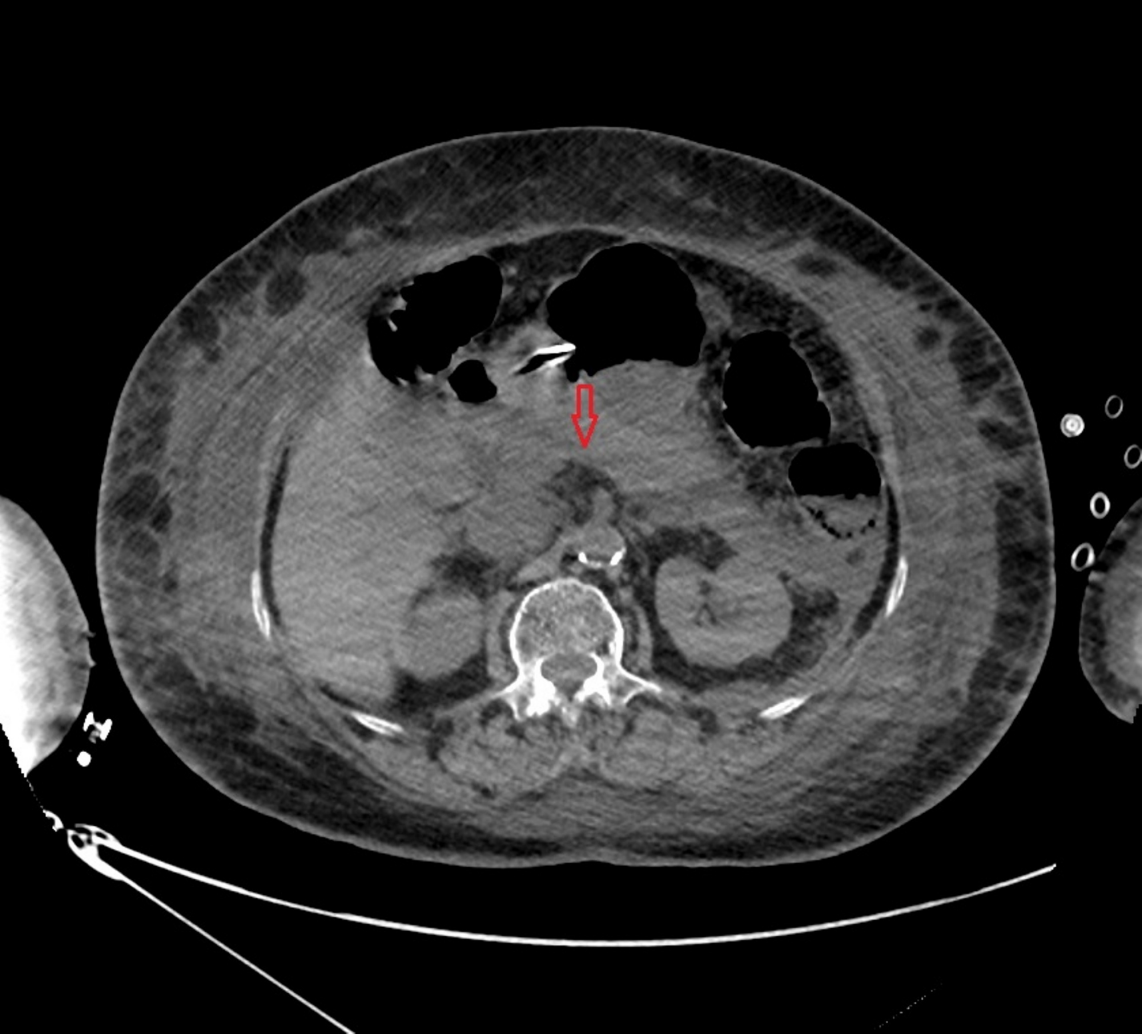
Computerized tomography (CT) scan of the abdomen revealing an enlarged pancreas with inflammatory fluid surrounding it, consistent with acute pancreatitis

On laboratory tests, the patient was found to have an elevated lipase of 11,916 IU/dL and lactic acid of 5.7 mmol/L otherwise with normal complete blood count, basic metabolic profile and liver function tests. Computerized tomography (CT) scan of the abdomen was performed and showed an enlarged pancreas with inflammatory fluid surrounding it, consistent with acute pancreatitis along with renal calcifications and diverticulosis (Figure 1).

The patient was admitted to the Intensive Care Unit (ICU) with an impression of acute pancreatitis. In the ICU, the patient was started on Ringer’s Lactate at 150 ml/hr and was kept Nil Per Os (NPO). On day 2, the patient remained hemodynamically stable, but still exhibited left upper quadrant and epigastric tenderness. Bowel sounds were decreased on physical examination. Additional tests were ordered to identify the etiology of acute pancreatitis. As a part of the workup, hypertriglyceridemia was not observed. Ultrasound of the right upper quadrant did not reveal gallstones. The patient was first diagnosed as having acute pancreatitis without end organ dysfunction and of an unclear etiology.

The patient continued to receive intravenous hydration with Ringer’s Lactate and serial abdominal examinations. Electrolytes, renal function, and liver function tests were normal and closely monitored. Although the patient had normal calcium levels, hypercalcemia remained still a highly probable explanation because of the renal calcifications found on CT. Serum calcium may have not reflected hypercalcemia, however, because in acute pancreatitis the calcium can be precipitated as a soap leading to hypocalcemia or hemoconcentration normally leading to hypercalcemia, which in this case did not happen and in contrary patient is normocalcemic. Thus, to determine if the patient was truly hypercalcemic, ionized calcium levels were drawn and found to be 5.27 mg/dL.

**Figure 2:**
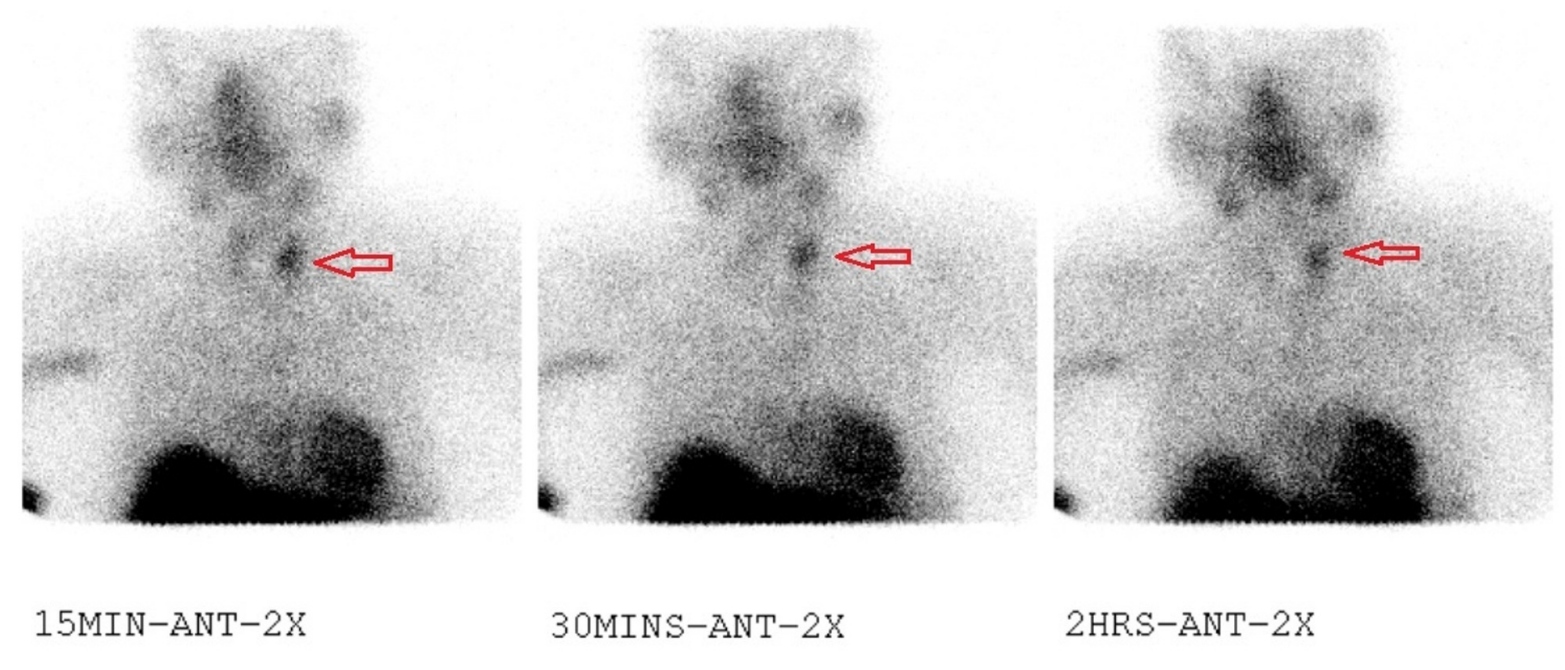
Scintigraphy revealing the presence of an adenoma on the left parathyroid gland

Parathyroid hormone (PTH) was found to be elevated at 210 pg/ml. To assess for secondary hyperparathyroidism, 25-OH Vitamin D level was measured and found to be normal. As a result, the patient was diagnosed with severe acute pancreatitis secondary to hypercalcemia, likely due to primary hyperparathyroidism. A Sestamibi parathyroid scintigraphy was performed to identify the underlying etiology of the patient’s hyperparathyroidism. The scintigraphy revealed the presence of an adenoma on the left parathyroid gland (Figure 2).

The patient’s Bedside Index for Severity in Acute Pancreatitis (BISAP) score was 4, predicting a 13%-19% mortality rate. Her hypercalcemia was aggressively managed to best improve her prognosis, and endocrinology recommended to start 30 mg Cinacalcet. As the patient’s hypercalcemia was treated, patient improved clinically and her lipase trended downward from 11,916 IU/dL to 59 IU/dL, further confirming this as the unusual but ultimate cause of the acute pancreatitis.  Patient eventually recovered from acute pancreatitis and was able to tolerate diet. Ear, Nose and Throat (ENT) service was consulted for possible parathyroidectomy. Patient and the family agreed for parathyroidectomy and is planned in the near future.

## Discussion

A diagnosis of primary hyperparathyroidism (PHPT) is characterized by excessive secretion of parathyroid hormone, which then leads to hypercalcemia and hypercalciuria. The most common cause of primary hyperparathyroidism is single or multiple adenomas (80-85%), as was the case with the patient we describe here. However, parathyroid hyperplasia (15-20%) and parathyroid carcinoma (<1%) can also produce primary hyperparathyroidism. On average, parathyroid adenomas remain less than 1 g in weight and 2 cm in size, which helps explain why the gold standard treatment for PHPT is to remove the gland in question through a parathyroidectomy [4].

The association between primary hyperparathyroidism and acute pancreatitis is not especially strong; however, it is an association that can be attributed to the role of hypercalcemia in PHPT pathogenesis. On a mechanistic level, it is thought that increased PTH leads to hypercalcemia, which then results in calcium deposits within pancreatic ducts. These deposits activate pancreatic enzymes, thereby inducing acute pancreatitis [3].

In a retrospective study examining 61 patients hospitalized for pancreatitis (54 patients with acute pancreatitis and five patients with chronic pancreatitis), only five patients were ultimately found to have PHPT (8%). These five patients were all female and between the ages of 40-76 years, with a mean age of 54 years. This could be indicative of the fact that a particular patient demographic is more susceptible to PHPT or it could also be a purely coincidental observation. Either way, this case series illustrated that longstanding PHPT may first be revealed by an episode of acute pancreatitis [5].

The previous study assessed the statistical likelihood of identifying PHPT in patients who have already been diagnosed with pancreatitis. Similarly, another study involving 100 patients examined the reverse scenario. This study determined the likelihood of identifying pancreatitis in patients who have already been diagnosed with PHPT. It found that although 52% of patients with PHPT complained of gastrointestinal symptoms, only 3% suffered from acute pancreatitis. This study also concluded that PHPT is often detected too late, for 35% of cases are diagnosed more than 10 years after the first appearance of a characteristic symptom. This statistic speaks to the importance of viewing pancreatitis, especially in the absence of an obvious etiology, as a potential bellwether of PHPT [6].

A literature review reveals past case reports of patients who received a diagnosis of acute pancreatitis in conjunction with one of primary or secondary hyperparathyroidism. After all, those with PHPT are at 10 times greater a risk of developing pancreatitis than the general population [7]. In four previous cases, patients were diagnosed by computed tomography, cervical ultrasound, and Sestamibi parathryoid scintigraphy as having parathyroid adenomas. Three of the four patients experienced complications (two instances of pseudocysts and one of pancreatic necrosis), but none of them had recurrent bouts of pancreatitis after being receiving treatment [8].

In one instance, the first sign of a patient’s primary hyperparathyroidism was acute pancreatitis, which resolved within ten days of reducing the patient’s serum calcium level with medical therapy [9]. In another instance, a 66-year-old woman, who had been treated for acute pancreatitis twice, was found to have serum calcium of 12.4 mg/dL and a PTH of 253 pg/dL. A soft tissue mass was identified within the left lobe of her thyroid by ultrasonography and computed tomography of the neck, and a Sestamibi parathyroid scintigraphy showed an accumulation of radiotracer uptake within this mass. Although the pathology report indicated no signs of invasion, a left hemithyroidectomy was performed to remove the parathyroid adenoma and restore normal calcium and PTH levels [10].

The case we present here is a particularly interesting one because it demonstrates the unusual systemic effects of an already rare endocrine disorder. Furthermore, it illustrates how pancreatitis can actually predate the diagnosis of underlying parathyroid adenoma or carcinoma. Thus, clinicians must be cognizant of primary hyperparathyroidism when encountering non-biliary, non-alcoholic acute pancreatitis in the context of hypercalcemia.

## Conclusions

Acute pancreatitis as a result of primary hyperparathyroidism is a rare clinical occurrence, one with an estimated prevalence rate of 1.5-7%. Despite this fact, it is important for clinicians to be aware of the connection between these two conditions, for acute pancreatitis can pose significant risks of morbidity and mortality. Although uncommon, the association between acute pancreatitis and primary hyperparathyroidism can be explained by the fact that hyperparathyroidism produces hypercalcemia. Hypercalcemia can then lead to calcium deposits within the pancreas, which activate pancreatic enzymes, thereby inducing acute pancreatitis. While acute pancreatitis is uncommonly due to primary hyperparathyroidism, a diagnosis of acute pancreatitis in many instances predates one of primary hyperparathyroidism. For this reason, it is crucial to consider hyperparathyroidism when encountering patients with non-biliary, non-alcoholic acute pancreatitis in the context of hypercalcemia.
